# Complicated Gallstones after Laparoscopic Sleeve Gastrectomy

**DOI:** 10.1155/2014/468203

**Published:** 2014-07-03

**Authors:** Eleni Sioka, Dimitris Zacharoulis, Eleni Zachari, Dimitris Papamargaritis, Ourania Pinaka, Georgia Katsogridaki, George Tzovaras

**Affiliations:** Department of Surgery, University Hospital of Larissa, Viopolis, 41110 Larissa, Greece

## Abstract

*Background.* The natural history of gallstone formation after laparoscopic sleeve gastrectomy (LSG), the incidence of symptomatic gallstones, and timing of cholecystectomy are not well established.* Methods.* A retrospective review of prospectively collected database of 150 patients that underwent LSG was reviewed.* Results.* Preoperatively, gallbladder disease was identified in 32 of the patients (23.2%). Postoperatively, eight of 138 patients (5.8%) became symptomatic. Namely, three of 23 patients (13%) who had evident cholelithiasis preoperatively developed complicated cholelithiasis. From the cohort of patients without preoperative cholelithiasis, five of 106 patients (4.7%) experienced complicated gallstones after LSG. Total cumulative incidence of complicated gallstones was 4.7% (95% CI: 1.3–8.1%). The gallbladder disease-free survival rate was 92.2% at 2 years. No patient underwent cholecystectomy earlier than 9 months or later than 23 months indicating the post-LSG effect.* Conclusion.* A significant proportion of bariatric patients compared to the general population became symptomatic and soon developed complications after LSG, thus early cholecystectomy is warranted. Routine concomitant cholecystectomy could be considered because the proportion of patients who developed complications especially those with potentially significant morbidities is high and the time to develop complications is short and because of the real technical difficulties during subsequent cholecystectomy.

## 1. Introduction

The incidence of cholelithiasis has been reported to be 5% in the general population, while it is significantly increased in obese population reaching 45% [1–3]. After bariatric surgery, weight loss of more than 25% of the original weight is considered to be the only predictive factor to postoperative gallstone formation [4, 5].

The incidence of gallstone formation differs between the various types of bariatric procedures. Asymptomatic gallstones are reported in 26.5% in gastric banding patients [6], though only 6.8% of patients become symptomatic postoperatively [7]. In addition, asymptomatic gallstones ranged from 30 to 52.8% after 6 to 12 months postoperatively [8–10], whilst symptomatic gallstones occurred by 7–16% in gastric Roux-en-Y by pass (RYGB) patients [8, 10–12]. Despite that, cholecystectomy after RYGB was necessary only for 3.9–17.6% of the patients whether or not stones were present before bariatric surgery [13].

Laparoscopic cholecystectomy (LC) in bariatric patients may be technically challenging due to suboptimal port placement and difficult body habitus. Furthermore, it is accompanied by potential risks such as lengthening of operative time, increased morbidity, and prolonged hospitalization. Serious complications have been reported as high as 2% to 3% of cases [14].

The published data are not illuminating in laparoscopic sleeve gastrectomy (LSG). To the best of our knowledge, only few case series exist in the literature. Moreover, there is lack of protocols concerning the management of gallstones after LSG. It seems that current policy is relied on local institution practice. Besides, the setting of cholecystectomy in relation to LSG as routine, selective, simultaneous, or delayed remains an ongoing therapeutic dilemma.

The aim of this study was a retrospective analysis of our prospectively collected data in order to determine the incidence of complicated gallstone disease after LSG.

## 2. Materials and Methods

The prospectively collected database of the morbidly obese patients who underwent LSG between August 2006 and December 2011 in our academic centre was reviewed. Medical records and histopathologic data were also reviewed.

Eligibility for surgery was defined according to the 1991 NIH consensus criteria for bariatric surgery [15]. Exclusion criteria were heavy sweaters, patients with suspected gastroesophageal reflux disease, as suggested by severe symptoms and endoscopic findings, patients with psychiatric disorders and addiction to either drugs or alcohol, and patients with high operative risk. The operative technique has been previously described [16].

Transabdominal ultrasound (US) was performed in all patients preoperatively to rule out gallstones or sludge. According to the protocol, patients with positive findings on ultrasound were counselled for concomitant laparoscopic cholecystectomy. Patients in the preoperative appointments were informed of the evidence of cholelithiasis and the potential risks and benefits of the arrangement of two procedures. The authors adopted the elective approach, meaning that simultaneous cholecystectomy was performed in symptomatic patients. Laparoscopic cholecystectomy was performed at the beginning of the procedure with the placement of an extra trocar. Postoperative prescription of ursodeoxycholic acid was not practiced in our management protocol.

Postoperative follow-up was performed at 2 weeks, 1 month, 3 months, 6 months, and 1 year and then yearly postoperatively. Patients were interviewed in follow-up appointments and complications related to gallbladder disease were recorded. Patients in this series were followed up for at least six months postoperatively.

## 3. Statistical Analysis

Statistical analyses were performed using the software SPSS 19 (SPSS Inc., Chicago, IL, USA) and Stata 11 (StataCorp. 2009, College Station, TX, USA). Quantitative variables were presented as means ± standard deviation or median with interquartile range or range. Qualitative data were presented as absolute frequencies and proportions. Prevalence, cumulative incidence, and corresponding confidence interval (95% CI) were calculated. Incidence per each interval was also calculated using life tables based on the actuarial method. Kaplan-Meier estimator was used to estimate survival rates and the corresponding 95% confidence intervals (type log-log) after LSG operation providing a Kaplan-Meier survival estimate plot.

## 4. Results

During the entire study period, one hundred sixty-five consecutive patients underwent LSG. Gallbladder follow-up data were obtained for 150 patients (92.6%). The median age was 40 years (range 18–62) and the median BMI was 46.1 (range 35–61). Patients in this study were predominantly female (79%). The median follow-up was 26 months (range 1–62).

Prior cholecystectomy was performed in 12 patients (8%). Preoperatively, positive gallbladder disease was identified in 32 patients (23.2%). In detail, pathologic findings were gallstones in 29 patients and sludge in 3 patients. Therefore, preoperative evidence of gallbladder disease was shown in 31.2%.

Simultaneous cholecystectomy was performed in 9 of 32 patients who had preoperative gallstones and were symptomatic. Eight operations were completed laparoscopically, while one open cholecystectomy was performed due to multiple adhesions from previous laparotomy. Neither perioperative or postoperative complications occurred.

**Figure 1:**
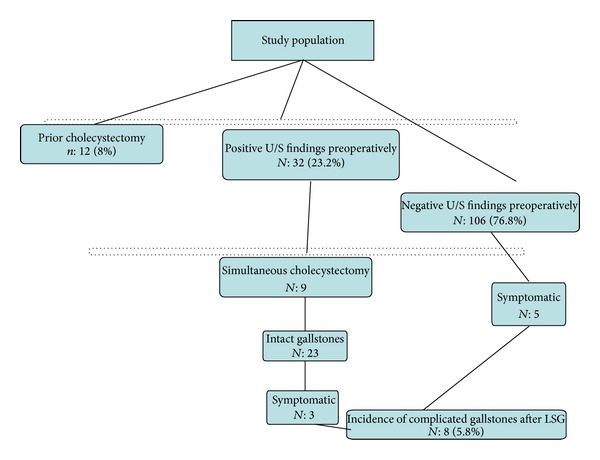
Incidence of complicated gallstones after LSG.

Thus, 23 patients left the operating room with intact gallstones. Of these, three patients required cholecystectomy eventually. These patients presented at 9, 23, and 15 months after LSG with acute cholecystitis, biliary colic, and pancreatitis, respectively. The postoperative period was uneventful. To the contrary, negative ultrasound findings were observed in 106 patients. Five patients of this group, with no evidence of gallstone disease preoperatively, presented with complicated gallstones. Three patients presented with acute cholecystitis and two patients suffered from choledocholithiasis. Thus, incidence of complicated gallstones postoperatively was estimated at 5.8% (Figure 1).

**Figure 2:**
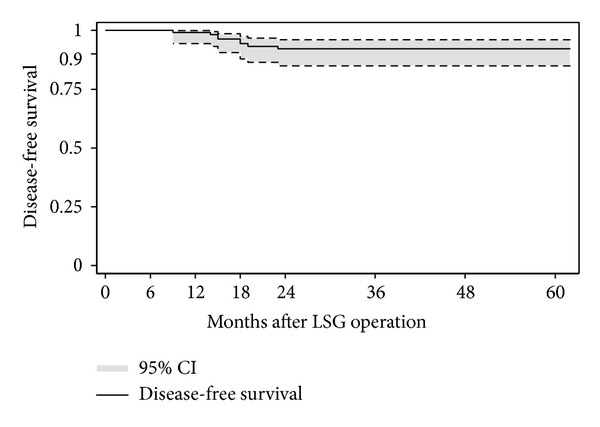
Kaplan-Meier survival estimate plot. Kaplan-Meier analysis of patients subsequently requiring laparoscopic cholecystectomy after LSG due to symptomatic cholelithiasis. The biliary complication-free survival rates were 99.2% (95% CI, 94.4–99.9%) at 12 months, 94.4% (95% CI, 87.9–97.4%) at 18 months, and 92.2% (95% CI, 85.0–96.0%) at 24 months.

All patients were diagnosed at intervals specified in Table 1. No late complications were noted. All patients except for one were managed with surgical intervention. Consequently, post-LSG cholecystectomy was performed in 7 patients whether or not preoperative gallstones were detected. Total cumulative incidence of cholecystectomy was 4.7% (95% CI: 1.3–8.1%). Kaplan-Meier analysis detected that the biliary complication-free survival rates were 99.2% (95% CI, 94.4–99.9%) at 12 months, 94.4% (95% CI, 87.9–97.4%) at 18 months, and 92.2% (95% CI, 85.0–96.0%) at 24 months after LSG (Figure 2).

## 5. Discussion

There is a paucity of data regarding preoperative evidence of gallstones, incidence of cholelithiasis with concomitant complications, and gallstone formation after LSG. In the literature, prior cholecystectomy in patients scheduled for bariatric surgery was anticipated at percentages of 11–23% [13]. In particular for LSG, Li et al. reported a percentage of 32.79% [17]. Our results are similar with these studies, since 23.2% of our patients were defined with preoperative gallbladder disease and previous cholecystectomy was performed in 8% of our patients.

In our series, one patient experienced complicated gallstones during the first postoperative year, while the other cases appeared during the second postoperative year. That implies what happened during the period of rapid weight loss. Overall, no patient underwent cholecystectomy earlier than 9 months or later than 23 months after LSG. That indicates the post-LSG effect regarding gallstones. It seems that this effect is similar to the effect of RYGB, since the gallstones tend to occur in the first 6–12 months and rarely after 2 years [18].

Although 23 patients were at risk for complicated gallstones due to preoperative evidence of gallstones, only three patients became symptomatic and required cholecystectomy. Thus, the risk for this group was 13.04%. On the other hand, the risk for the patients without preoperative gallstones was 4.7%. In detail, acute cholecystitis was diagnosed in 4 patients, biliary colic in 1 patient, choledocholithiasis in 2 patients, and pancreatitis in 1 patient. Our data are consistent with other series. More specifically, Tucker et al. reported symptomatic cholelithiasis and choledocholithiasis in 2 and 1 patients, respectively, in a total of 164 patients (1, 8%) [19]. Arias et al. reported that a percentage of 3.8% of patients developed symptomatic gallstones postoperatively, while 1.8% had symptoms of gallstones prior to surgery [20]. Li et al. showed that 3.8% of patients after LSG developed symptomatic gallstones requiring medical attention and surgical intervention [17]. Lalor et al. mentioned choledocholithiasis in 0.7% [21]. Uglioni et al. reported 1 case of acute cholecystitis and 2 cases of cholelithiasis (3.8%) [22].

Nowadays, the conservative regimen of reserving cholecystectomy for symptomatic disease in gastric banding and RYGB serves as a safe modality of treatment [7, 23], while asymptomatic gallstones (silent gallstones) represent a dilemmatic approach. The natural history of asymptomatic gallstones suggests that many affected individuals will remain asymptomatic [24, 25]. Furthermore, recent trend analysis in RYGB patients suggests that concomitant cholecystectomy should be considered only in symptomatic gallstones [26].

The current statement of cholecystectomy and LSG has not been validated. Three options could be available. The first is the offer of laparoscopic cholecystectomy, whether gallstones are identified in the routine preoperative assessment, even if they are asymptomatic (approach of Hamad) [27]. This prophylactic approach presupposes that natural history of gallbladder disease in LSG patients is different than that in general population. The second is the simultaneous service of cholecystectomy with LSG, without preoperative investigation (approach of Fobi) [8]. The third is the treatment of the symptomatic patients only without preoperative screening (noninterventionist policy) [7]. However, no standard of care regarding the preoperative work-up or even postoperative care has been established. In our practice, preoperative transabdominal ultrasound was obtained for all patients. Furthermore, the authors' philosophy was to perform elective cholecystectomy in patients with preoperative evidence of gallbladder disease that were symptomatic. However, the fact that eight of 138 patients (5.8%) became symptomatic and soon developed complications warrants the recommendation for early cholecystectomy. Furthermore, a significant proportion of bariatric patients compared to the general population developed complications in the absence or not of preoperative gallstones. As a consequence, routine concomitant cholecystectomy could be considered because the proportion of patients who developed complications especially those with potentially significant morbidities, such as choledocholithiasis, cholangitis, and pancreatitis, are high and the time to develop complications is short and because of the real technical difficulties during subsequent cholecystectomy. Nevertheless, the formulating policy regarding the investigation and management of cholelithiasis in LSG as a part of the routine assessment and care of the bariatric patient needs to be further evaluated.

Regarding the management, all cases except for one were surgically managed. From a technical point of view, the cholecystectomy after LSG is not technically straightforward due to trocar placement and body habitus. Thus, the position of trocars made the performance of cholecystectomy more difficult than it would be expected. Additional trocar was inserted to improve access. On the other hand, the setting of cholecystectomy after LSG has the advantage that the different body habitus and the fact that the patient had lost weight facilitated the cholecystectomy.

The use of ursodeoxycholic acid has been proposed as a preventive measure for the gallstone formation. More specifically, Sugerman et al. reported that the oral dose of 600 mg ursodiol following gastric bypass for 6 months or even until gallstone formation was associated with decreased rate of gallstone formation [28]. These results are also in compliance with another study in vertical banded gastroplasty and gastric banding, which also supported that the rate of cholecystectomy was less frequent in the group receiving ursodiol compared to placebo group (4.7 versus 12%) [29]. Mc et al., in a meta-analysis, concluded that rate of gallstone formation was reduced by the protective use of ursodiol therapy [30]. However, recent cost-effective analysis reported that even though the use of ursodeoxycholic acid lessened the costs of concurrent cholecystectomy and reduced the hospital stay along with logical cost raise in selective cholecystectomy, the authors concluded that the prescription of ursodiol is unaffordable as an additional cost and proposed the nonuse of ursodiol after bariatric surgery [31].

Some limitations of our study should be acknowledged. The retrospective nature of our study and the sample size should be taken into account. Additionally, we did not perform postoperative ultrasound to evaluate the real rate of gallstone formation after LSG. However, we provide a series which relies on prospectively collected data. We also estimate time-dependent gallbladder disease-free survival rates. Furthermore, we describe the natural history of gallstones until the mid-term period. Possibly, these may change in the long-term evaluation.

## 6. Conclusion

A significant proportion of bariatric patients (5.8%) compared to the general population became symptomatic and soon developed complications in the absence or not of preoperative gallstones after LSG; thus, recommendation for early cholecystectomy is warranted. Routine concomitant cholecystectomy could be considered because the proportion of patients who developed complications especially those with potentially significant morbidities are high and the time to develop complications is short and because of the real technical difficulties during subsequent cholecystectomy.

## Figures and Tables

**Table 1:** Incidence of cholecystectomy at intervals after LSG (laparoscopic sleeve gastrectomy).

Interval after LSG (months)	Number of patients entering this interval	Number of patients withdrawing during interval	Number of patients with postoperative ultrasounds	Number of patients exposed to risk^#^	Number of patients who underwent cholecystectomy	Incidence per interval (cumulative incidence)	Total cumulative incidence (95% CI)
0	138	0	0	138	0	0.0%	7/138 5.1% (1.4%–8.7%)
<3	138	11	0	132.5	0	0.0%	
3 to <6	127	11	0	121.5	0	0.0%	
6 to <12	116	9	1	111.5	1	0.9%	
12 to <24	106	27	7	92.5	6	6.5%	
24+	73	73	0	36.5	0	0.0%	

^#^Patients exposed to risk = Patients entering – (1/2) ∗  Patients withdrawing.
